# Supplementary material for: SMARCA4 Mutations in Gastroesophageal Adenocarcinoma: An Observational Study via a Next-Generation Sequencing Panel
Source: Cancers (Basel). 2024 Mar 27;16(7):1300. doi: 10.3390/cancers16071300 (PMC11010836; doi:10.3390/cancers16071300)
Supplement: Supplementary file 1 [file cancers-16-01300-s001.zip › cancers-2895710-supplementary.pdf]

Supplementary Table S1. List of genes detected by the NGS panel

AKT1	BTK	CREBBP	FGF19	HRAS	MAPK1	NBN	PIK3CB	RAF1	SPOP
AKT2	CBL	CSF1R	FGF3	IDH1	MAX	NF1	PIK3R1	RB1	SRC
AKT3	CCND1	CTNNB1	FGFR1	IDH2	MDM2	NF2	PMS2	RET	STAT3
ALT	CCND2	DDR2	FGFR2	IGF1R	MDM4	NFE2L2	POLE	RHEB	STK11
AR	CCND3	EGFR	FGFR3	JAK1	MED12	NOTCH1	PPARG	RHOA	TERT
ARAF	CCNE1	ERBB2	FGFR4	JAK2	MET	NOTCH2	PPP2R1A	RICTOR	TOP1
ARID1A	CDK12	ERBB3	FLT3	JAK3	MLH1	NOTCH3	PTCH1	RNF43	TP53
ATM	CDK2	ERBB4	FOXL2	KDR	MRE11A	NRAS	PTEN	ROS1	TSC1
ATR	CDK4	ERCC2	GATA2	KIT	MSH2	NTRK1	PTPN11	SETD2	TSC2
ATRX	CDK6	ESR1	GNA11	KNSTRN	MSH6	NTRK2	RAC1	SF3B1	U2AF1
AXL	CDKN1B	EZH2	GNAQ	KRAS	MTOR	NTRK3	RAD50	SLX4	XPO1
BAP1	CDKN2A	FANCA	GNAS	MaGOH	MYC	PALB2	RAD51	SMAD4	
BRAF	CDKN2B	FANCD2	H3F3A	MAP2K1	MYCL	PDGFRA	RAD51B	SMARCA4	
BRCA1	CHEK1	FANCI	HIST1H3B	MAP2K2	MYCN	PDGFRB	RAD51C	SMARCB1	
BRCA2	CHEK2	FBXW7	HNF1A	MAP2K4	MYD88	PIK3CA	RAD51D	SMO	

Supplementary Table S2. Coverage by gene and codon(s) tested for adequate amplicons

AKT1 (NM_005163)	3 (16-56), 4 (59-96), 6 (146-149), 6-7 (177-195), 9 (235-274), 11 (320-380), 12 (391-411), 13 (450-455), 14 (459-481)
AKT2 (NM_001626)	3 (16-55), 4 (76-96), 6 (148-153), 7 (192-210), 8 (221-236), 10 (278-279), 11 (322-381), 12 (395-421), 14 (456-482)
AKT3 (NM_005465)	2 (16-28), 2 (48-58), 3 (66-95), 4 (123-143), 6 (188-209), 8 (233-267), 10 (317-367), 11 (388-399)
ALK (NM_004304)	20-28 (1096-1388)
AR (NM_000044)	1 (1-45), 1 (135-178), 1 (245-285), 1 (342-388), 3 (615-629), 4 (707-725), 6-7 (797-831), 8 (870-910)
ARAF (NM_001654)	7 (186-216)
ARID1A (NM_006015)	1 (28-73), 1 (106-151), 1-8 (161-838), 8-20 (861-2062), 20 (2081-2286)
ATM (NM_000051)	2 (1-24), 3-5 (40-166), 6-9 (174-412), 10-14 (431-724), 14-17 (740-865), 18-19 (880-974), 20-25 (1001-1249), 26(1258-1283), 26-30 (1292-1537), 31-35 (1550-1773), 36-40 (1790-1992), 41-44 (2003-2136), 44-46 (2146-2262), 47-49(2270-2389), 49-51 (2414-2542), 52-54 (2544-2667), 55 (2671-2711), 56-57 (2718-2806), 58-63 (2823-3028), 63(3041-3057)
ATR (NM_001184)	1-2 (1-47), 3-5 (51-450), 6-7 (458-578), 8 (584-629), 9-10 (636-781), 11-13 (783-882), 13-15 (896-1052), 16 (1058-1119), 17-19 (1125-1237), 21-23 (1274-1416), 24-27 (1423-1618), 28-29 (1628-1692), 29-34 (1705-1965), 35-38 (1967-2184), 39 (2187-2212), 40 (2230-2296), 41 (2300-2347), 43-47 (2398-2645)
ATRX (NM_000489)	1-2 (1-45), 4 (64-81), 6 (124-135), 6-8 (152-221), 9 (257-312), 9 (329-571), 9 (595-679), 9 (738-794), 9 (809-1148), 9-10 (1184-1270), 11 (1279-1301), 12 (1315-1374), 13 (1380-1405), 15 (1440-1519), 16 (1525-1562), 17-18 (1570-1644), 19(1653-1712), 20-21 (1718-1766), 21 (1785-1807), 23 (1860-1899), 24-26 (1908-1987), 28 (2073-2076), 29 (2117-2138), 31 (2234-2283), 32 (2288-2325), 33 (2353-2357), 34-35 (2364-2493)
AXL (NM_001699)	1 (1-29), 3 (103-137), 6 (223-261), 7 (288-332), 8 (351-378), 9 (386-429), 11 (473-497), 12 (516-536), 15 (593-633), 17(670-686), 19 (769-782)
BAP1 (NM_004656)	1-17 (6-730)
FGFR1 (NM_015850)	4 (120-148), 6 (217-247), 8 (311-355), 9 (408-426), 12 (516-553), 14 (642-657), 16 (709-727), 18 (786-821)
FGFR2 (NM_000141)	6 (209-235), 7 (250-273), 7-8 (277-350), 9 (362-399), 10 (453-480), 12 (521-558), 13 (589-621), 14-15 (631-686), 16 (699-730), 18 (768-804)
FGFR3 (NM_000142)	2 (1-37), 4 (127-133), 8 (329-359), 9 (368-412), 10 (423-439), 14 (633-653), 15-16 (659-719)
FGFR4 (NM_022963)	1 (1-31), 2 (72-116), 4 (163-199), 6 (243-283), 8 (359-392), 10-11 (467-535), 13 (609-632), 15 (683-713)
FLT3 (NM_004119)	3 (93-123), 6 (205-213), 8 (302-346), 10 (402-435), 12 (502-533), 15 (613-648), 18 (742-764), 20-21 (808-885), 24 (957-994)
FOXL2 (NM_023067)	1 (94-137)
GATA2 (NM_032638)	4 (291-330), 5 (341-379), 6 (452-481)

GNA11 (NM_002067)	4 (165-196), 5 (205-240)
GNAQ (NM_002072)	2 (60-100), 4-5 (163-224)
GNAS (NM_000516)	8 (196-218), 9 (220-240)
H3-3A (NM_002107)	2 (1-36)
H3C2 (NM_003537)	1 (6-37), 1 (61-102)
HNF1A (NM_000545)	2 (109-116), 3 (196-238)
HRAS (NM_005343)	2 (6-34), 3 (44-87), 4 (97-150)
IDH1 (NM_005896)	4 (100-138)
IDH2 (NM_002168)	4 (125-155), 4 (162-178)
IGF1R (NM_000875)	1 (1-32), 2 (138-176), 4 (318-328), 6 (416-457), 8 (572-608), 10 (674-711), 12 (829-865), 14 (928-946), 17 (1063-1099), 20 (1196-1220)
JAK1 (NM_002227)	14 (634-663), 15-16 (668-743)
JAK2 (NM_004972)	14 (600-622)
JAK3 (NM_000215)	11-12 (502-563), 15 (639-683)
KDR (NM_002253)	11 (482-512), 16 (766-791), 23 (1026-1064), 24 (1082-1102)
KIT (NM_000222)	8 (411-445), 9 (487-514), 10-20 (516-934)
KNSTRN (NM_033286)	1 (1-28)
KRAS (NM_004985)	2-3 (1-93), 4-5 (97-189)
MAGOH (NM_002370)	5 (114-147)
MAP2K1 (NM_002755)	2 (27-96), 3 (98-135), 6 (190-227), 11 (357-394)
MAP2K2 (NM_030662)	3 (102-123)
MAP2K4 (NM_003010)	4 (132-154), 5 (178-208)
MAPK1 (NM_002745)	7 (300-322)
MAX (NM_002382)	3 (22-56), 4 (58-83)
MDM2 (NM_002392)	2 (22-33), 3 (51-58), 4 (79-103), 7 (160-175), 8 (198-228), 9 (236-267), 10 (289-306), 11 (340-373), 11 (406-439)
MDM4 (NM_002393)	2 (1-17), 3 (27-50), 5-6 (96-137), 7 (158-171), 8 (212-224), 9 (229-269), 11 (302-334), 11 (348-386), 11 (428-459)
MED12 (NM_005120)	2 (34-59), 26 (1199-1227)
MET (NM_001127500)	2 (151-192), 2 (217-257), 2 (287-331), 2 (344-384), 14 (981-989), 14-21 (1003-1367)
MLH1 (NM_000249)	1-11 (1-346), 12-19 (353-757)
MRE11 (NM_005590)	2 (1-7), 3-6 (20-182), 7 (190-220), 8-11 (226-398), 12-19 (409-681)
MSH2 (NM_000251)	1-3 (1-215), 4 (228-259), 5 (265-307), 6-9 (315-504), 11-12 (554-661), 13-15 (669-852), 16 (879-935)
MSH6 (NM_000179)	1-10 (1-1361)
MTOR (NM_004958)	29 (1418-1443), 30 (1452-1490), 31 (1509-1524), 39 (1789-1803), 40 (1876-1905), 43 (1971-1994), 43-44 (1999-2035), 47-48 (2187-2251), 53 (2394-2434), 56-57 (2483-2520)
MYC (NM_002467)	1-2 (1-87), 2 (98-131), 2 (140-185), 2 (196-226), 3 (268-278), 3 (297-340), 3 (352-395), 3 (413-453)
MYCL (NM_005376)	2 (60-96), 2 (163-237)
MYCN (NM_005378)	2 (1-125), 2-3 (229-349), 3 (371-450)
MYD88 (NM_002468)	3 (181-221), 5 (259-269)
NBN (NM_002485)	1-5 (1-166), 6-11 (200-615), 13-16 (639-755)
NF1 (NM_001042492)	1-12 (1-464), 13-16 (483-596), 17-23 (616-1038), 25-29 (1071-1325), 30-32 (1346-1444), 33-58 (1447-2840)
NF2 (NM_000268)	1-5 (1-172), 7-10 (200-332), 11-16 (334-596)
NFE2L2 (NM_006164)	2 (23-59), 2 (74-104)
NOTCH1 (NM_017617)	2-27 (21-1680), 27 (1686-1723), 28-34 (1736-2116), 34 (2145-2556)
NOTCH2 (NM_024408)	1 (1-25), 3 (68-131), 4 (139-191), 4-31 (193-1878), 31-34 (1887-2472)
NOTCH3 (NM_000435)	1-2 (16-48), 3-18 (66-959), 18-24 (969-1292), 24 (1414-1449), 25-26 (1468-1631), 27-33 (1659-2153), 33 (2163-2322)
NRAS (NM_002524)	2 (1-22), 3 (49-89), 4 (110-150)
NTRK1 (NM_002529)	2 (71-92), 3 (106-120), 6 (192-227), 8 (321-393), 11 (418-446), 14 (553-599), 15 (658-682), 17 (747-790)
NTRK2 (NM_006180)	4 (1-43), 6 (96-120), 9 (197-239), 11 (294-366), 14 (433-466), 16 (490-531), 17 (580-588), 18 (601-641), 19 (674-723), 20 (725-756)
NTRK3 (NM_002530)	7 (155-161), 10 (303-318), 10 (377-402), 13 (432-456), 14 (472-518), 16 (576-630), 17 (662-711), 19 (808-826)
PALB2 (NM_024675)	1-3 (1-71), 4-6 (79-862), 7-13 (888-1187)
PDGFRA (NM_006206)	12-15 (552-719), 17-21 (775-960)

PDGFRB (NM_002609)	2 (1-14), 3 (99-122), 5 (211-235), 7 (333-370), 9 (450-456), 11 (527-545), 12-13 (587-638), 16 (753-782), 19 (881-900), 22 (969-1009), 23 (1093-1107)
PIK3CA (NM_006218)	2-3 (77-138), 5 (311-351), 8 (418-457), 10-11 (532-582), 14 (693-729), 21 (1015-1057)
PIK3CB (NM_006219)	1 (31-57), 3 (138-174), 5 (268-294), 7 (400-434), 10 (518-527), 11 (545-586), 14 (679-683), 16 (784-809), 20 (933-954), 22 (1026-1071)
PIK3R1 (NM_181523)	2-10 (1-433), 11-16 (440-725)
PMS2 (NM_000535)	1-2 (1-30), 2-5 (35-165), 6-11 (180-657), 12 (669-680), 12-13 (716-726), 15 (855-863)
POLE (NM_006231)	1-46 (1-2177), 47-49 (2217-2287)
PPARG (NM_015869)	1 (1-28), 2 (53-78), 3 (104-134), 3 (152-160), 4 (169-200), 5 (207-228), 5 (257-273), 6 (282-321), 6 (350-390), 7 (424-461)
PPP2R1A (NM_014225)	5 (172-199), 6 (220-263)
PTCH1 (NM_000264)	1-23 (6-1344), 23 (1374-1448)
PTEN (NM_000314)	1-4 (1-85), 5-7 (99-267), 8-9 (284-382)
PTPN11 (NM_002834)	3 (52-91), 13 (485-527)
RAC1 (NM_006908)	2 (18-36)
RAD50 (NM_005732)	1-5 (1-244), 6 (253-267), 7-8 (296-415), 9 (456-478), 10 (517-545), 11-12 (553-657), 13-14 (681-799), 15-16 (828-902),
	17 (907-917), 18 (944-967), 19 (987-1012), 21-23 (1055-1206), 24-25 (1212-1313)
RAD51 (NM_002875)	2-4 (1-115), 5-10 (121-340)
RAD51B (NM_133509)	2-5 (1-113), 5-6 (142-191), 7 (201-245), 8-11 (253-374)
RAD51C (NM_002876)	1-2 (1-136)
RAD51D (NM_133629)	1-7 (1-217)
RAF1 (NM_002880)	7 (235-276), 12 (407-442)
RB1 (NM_000321)	1-2 (1-85), 3 (89-127), 4 (130-159), 5-6 (167-194), 6-15 (196-464), 16 (480-500), 17-18 (523-582), 18-21 (587-736), 22-24 (743-835), 25-27 (841-929)
RET (NM_020975)	10-11 (609-654), 12-14 (713-869), 15-18 (875-1013)
RHEB (NM_005614)	2 (18-42)
RHOA (NM_001664)	2 (2-44)
RICTOR (NM_152756)	7 (153-175), 10 (291-297), 21 (658-684), 26 (834-863), 30 (981-1019), 31 (1210-1246), 32 (1382-1416), 35 (1588-1597), 38 (1690-1709)
RNF43 (NM_017763)	2-9 (1-442), 9-10 (455-784)
ROS1 (NM_002944)	36 (1926-1980), 38 (2002-2045), 39-40 (2050-2092), 40-42 (2106-2245)
SETD2 (NM_014159)	1-3 (1-549), 3-6 (567-1613), 7-11 (1624-1799), 12 (1809-1869), 12 (1911-2020), 14-21 (2037-2565)
SF3B1 (NM_012433)	14 (603-640), 14 (655-679), 15 (693-716), 15-16 (738-747)
SLX4 (NM_032444)	2-12 (1-1477), 12-15 (1493-1835)
SMAD4 (NM_005359)	9 (335-375), 12 (519-553)
SMARCA4 (NM_003072)	2-12 (1-644), 13-15 (648-756), 16-19 (759-936), 20-25 (954-1162), 26-35 (1183-1648)
SMARCB1 (NM_003073)	1-2 (1-72), 3-5 (78-206), 6-9 (210-386)
SMO (NM_005631)	3 (186-228), 4-5 (263-354), 6 (397-422), 9 (511-551), 11 (608-646)
SPOP (NM_003563)	5 (88-118), 6 (125-160)
SRC (NM_005417)	12 (374-418)
STAT3 (NM_139276)	13 (398-411), 20 (583-620), 21 (630-667)
STK11 (NM_000455)	1-8 (1-361), 9 (370-434)
TERT (NM_198253)	1 (1-18), 2 (267-310), 2 (394-425), 3 (553-585), 4 (626-650), 6 (718-762), 9 (823-852), 11 (920-948), 14 (1011-1043), 16 (1099-1133)
TOP1 (NM_003286)	20 (682-722)
TP53 (NM_000546)	2-11 (1-394)
TSC1 (NM_000368)	3-23 (1-1165)
TSC2 (NM_000548)	2-35 (1-1523), 37-42 (1555-1808)
U2AF1 (NM_006758)	2 (15-41), 6 (137-161)
XPO1 (NM_003400)	15 (563-575)
